# Noncontact Layer Stabilization of Azafullerene Radicals: Route toward High-Spin-Density Surfaces

**DOI:** 10.1021/acsnano.3c08717

**Published:** 2023-12-12

**Authors:** Yuri Tanuma, Gregor Kladnik, Luca Schio, Marion van Midden Mavrič, Bastien Anézo, Erik Zupanič, Gregor Bavdek, Ruben Canton-Vitoria, Luca Floreano, Nikos Tagmatarchis, Hermann A. Wegner, Alberto Morgante, Christopher P. Ewels, Dean Cvetko, Denis Arčon

**Affiliations:** † Jožef Stefan Institute, Jamova 39, SI-1000 Ljubljana, Slovenia; ‡ Center for Advanced Research of Energy and Materials (CAREM), Hokkaido University, Kita 13, Nishi 8, Kitaku, Sapporo 060-8628, Japan; § Faculty of Mathematics and Physics, University of Ljubljana, Jadranska 19, SI-1000 Ljubljana, Slovenia; ∥ CNR-IOM, Istituto Officina dei Materiali, Basovizza Area Science Park, 34149 Trieste, Italy; ⊥ Institut des Matériaux de Nantes Jean Rouxel (IMN), UMR 6502 CNRS, Nantes University, 44322 Nantes, France; # Faculty of Education, University of Ljubljana, Kardeljeva ploščad 16, SI-1000 Ljubljana Slovenia; ⊗ Theoretical and Physical Chemistry Institute, National Hellenic Research Foundation, 48 Vassileos Constantinou Avenue, Athens 11635, Greece; ¶ Institute of Organic Chemistry, Justus Liebig University Giessen, Heinrich-Buff-Ring 17, 35392 Giessen, Germany; ● Center for Materials Research (ZfM/LaMa), Justus Liebig University Giessen, Heinrich-Buff-Ring 16, 35392 Giessen, Germany; ○ Physics Department, University of Trieste, Via Valerio 2, 34012 Trieste, Italy

**Keywords:** azafullerene, radicals, qubit, scanning tunnelling microscopy, near edge X-ray absorption fine structure spectroscopy, density functional calculation

## Abstract

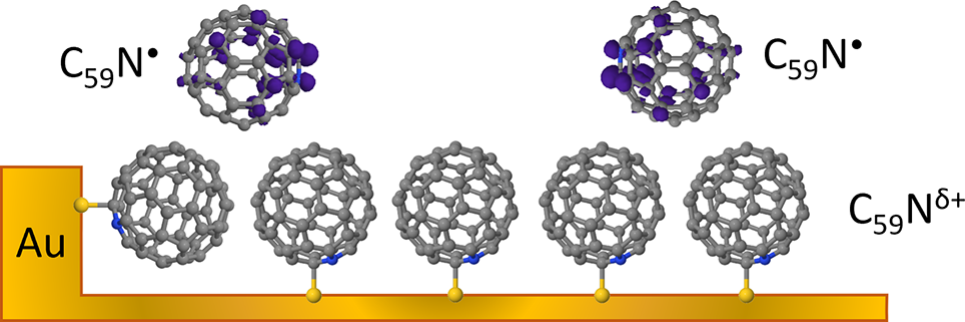

We deposit azafullerene C_59_N^•^ radicals in a vacuum on the Au(111) surface for layer thicknesses between 0.35 and 2.1 monolayers (ML). The layers are characterized using X-ray photoemission (XPS) and X-ray absorption fine structure (NEXAFS) spectroscopy, low-temperature scanning tunneling microscopy (STM), and by density functional calculations (DFT). The singly unoccupied C_59_N orbital (SUMO) has been identified in the N 1s NEXAFS/XPS spectra of C_59_N layers as a spectroscopic fingerprint of the molecular radical state. At low molecular coverages (up to 1 ML), films of monomeric C_59_N are stabilized with the nonbonded carbon orbital neighboring the nitrogen oriented toward the Au substrate, whereas in-plane intermolecular coupling into diamagnetic (C_59_N)_2_ dimers takes over toward the completion of the second layer. By following the C_59_N^•^ SUMO peak intensity with increasing molecular coverage, we identify an intermediate high-spin-density phase between 1 and 2 ML, where uncoupled C_59_N^•^ monomers in the second layer with pronounced radical character are formed. We argue that the C_59_N^•^ radical stabilization of this supramonolayer phase of monomers is achieved by suppressed coupling to the substrate. This results from molecular isolation on top of the passivating azafullerene contact layer, which can be explored for molecular radical state stabilization and positioning on solid substrates.

## Introduction

Organic radicals, organic compounds possessing one or more unpaired electrons in their electronic ground state, are usually thought of as transient or unstable species, since their unpaired electrons are highly reactive. However, their stability can be significantly improved by either sterically protecting the unpaired electron sites (for example with bulky side groups), or by increasing the delocalization of the unpaired electrons over several atoms.^1^ Such stable molecular radicals are, for example, considered as a simple platform to encode bits in the limits of quantum physics (qubits)^2^ or are a potential route for the design of synthetic and catalytic processes.^3^ One candidate for robust organic molecular radicals is azafullerene C_59_N,^4^ where one nitrogen replaces a carbon atom in the fullerene skeleton. Due to valence inequality between N and C, C_59_N is a closed-cage heterofullerene radical, where the odd electron resides on the carbon neighboring the substitutional nitrogen.^5^ However, due to their high reactivity, it readily forms closed shell dimers (C_59_N)_2_^5^ or C_59_HN^6^ in the bulk phase. The spin-active radical species can then be accessed by thermolysis or photolysis of the parent (C_59_N)_2_^7−11^ as the two C_59_N units are weakly bound by ∼0.78 eV, but they nevertheless show high reactivity to ultrarapid redimerization (under inert conditions) or oxidation (in ambient environment).

From the perspective of molecular qubit applications, any complex architecture of molecular qubits needs to be ultimately placed in some 2D network, and molecular spin manipulation on solid substrates appears to be the next necessary step.^12^ The stability of molecular spins then decisively relies on particular interaction with the surface and suppressed intermolecular coupling that prevents formation of nonmagnetic oligomer or polymer assemblies. Similar requirements may also be defined for the radical catalytic reactions. Multilayers of azafullerenes on solid substrates have been previously realized in ultrahigh vacuum (UHV) by thermal deposition of C_59_N^•^ monomers, but the formed layers have been found predominantly in their stable diamagnetic (C_59_N)_2_ dimer entities.^13^ Early studies of C_59_N^•^ on reactive Si(111)-(7 × 7) and Si(100)-(1 × 2) surfaces indicate that almost no dimerization takes place at submonolayer coverage, proving that azafullerene sublimes as C_59_N^•^ monomers by UHV thermal deposition, yet no direct spectroscopic evidence of the radical character of such monomers on Si was given.^14−16^

Similarly, C_59_N^•^ monomer stabilization was reported for azafullerene monolayers on Cu(111), where strong binding to the substrate was observed.^17^ In fact, significant charge transfer from Cu to C_59_N^•^ was reported for the contact layer,^17^ which stabilizes monomers over dimers on Cu(111) and at the same time completely quenches the radical character of the azafullerene monomers.

The choice of Au(111) is expected to provide a substrate with a sufficiently weak interaction to avoid covalent bonding with C_59_N^•^ and thus preserve the radical state of azafullerenes. In fact, for a similar organic system—1,2,4-benzotriazin-4-yl radical (Blatter radical)—the radical character of single Blatter molecules could be retained on Au(111), as proven by near edge X-ray absorption fine structure (NEXAFS) spectroscopy^18^ and scanning tunneling microscopy (STM) identification of Kondo resonance.^19^

Here, we present a comprehensive experimental and theoretical study of ultrathin azafullerene films deposited in UHV on the Au(111) surface. From X-ray photoemission (XPS) and NEXAFS spectroscopy, we determine the azafullerene coupling and the radical state in films up to 2 monolayers (ML) thickness. Supported by DFT results, we find the direct spectroscopic fingerprint of the C_59_N^•^ radical state in the nitrogen K-edge NEXAFS, as the occurrence of a singly unoccupied molecular orbital (SUMO) lying close to the Fermi level. Analysis of NEXAFS and the complementary low-temperature topographic STM images prove that initially C_59_N^•^ monomers form hexagonally packed islands where individual azafullerenes orient toward the Au(111) surface with their nonbonded carbon orbital neighboring the nitrogen. The radical character of C_59_N^•^ monomer films is elucidated from the analysis of NEXAFS and shakeup structures in the N 1s XPS peak, i.e., photoelectron energy losses due to highest occupied molecular orbital (HOMO) to lowest unoccupied molecular orbital (LUMO) excitations. Site selective charge transfer between C_59_N^•^ monomers and the Au substrate is found for the monolayer films, as the contact strength between monomers and the Au substrate varies within the azafullerene islands due to their lattice mismatch with Au(111). Finally, we discover a high-density radical phase for the C_59_N^•^ supramonolayer (ML+) phase at coverage between 1 and 2 ML, where C_59_N^•^ monomers are isolated on top of the first layer. These molecules exhibit the largest SUMO peak intensity in the NEXAFS spectra and give insight into the nature of efficient stabilization of molecular radicals and their spins.

## Results and Discussion

**Figure 1:**
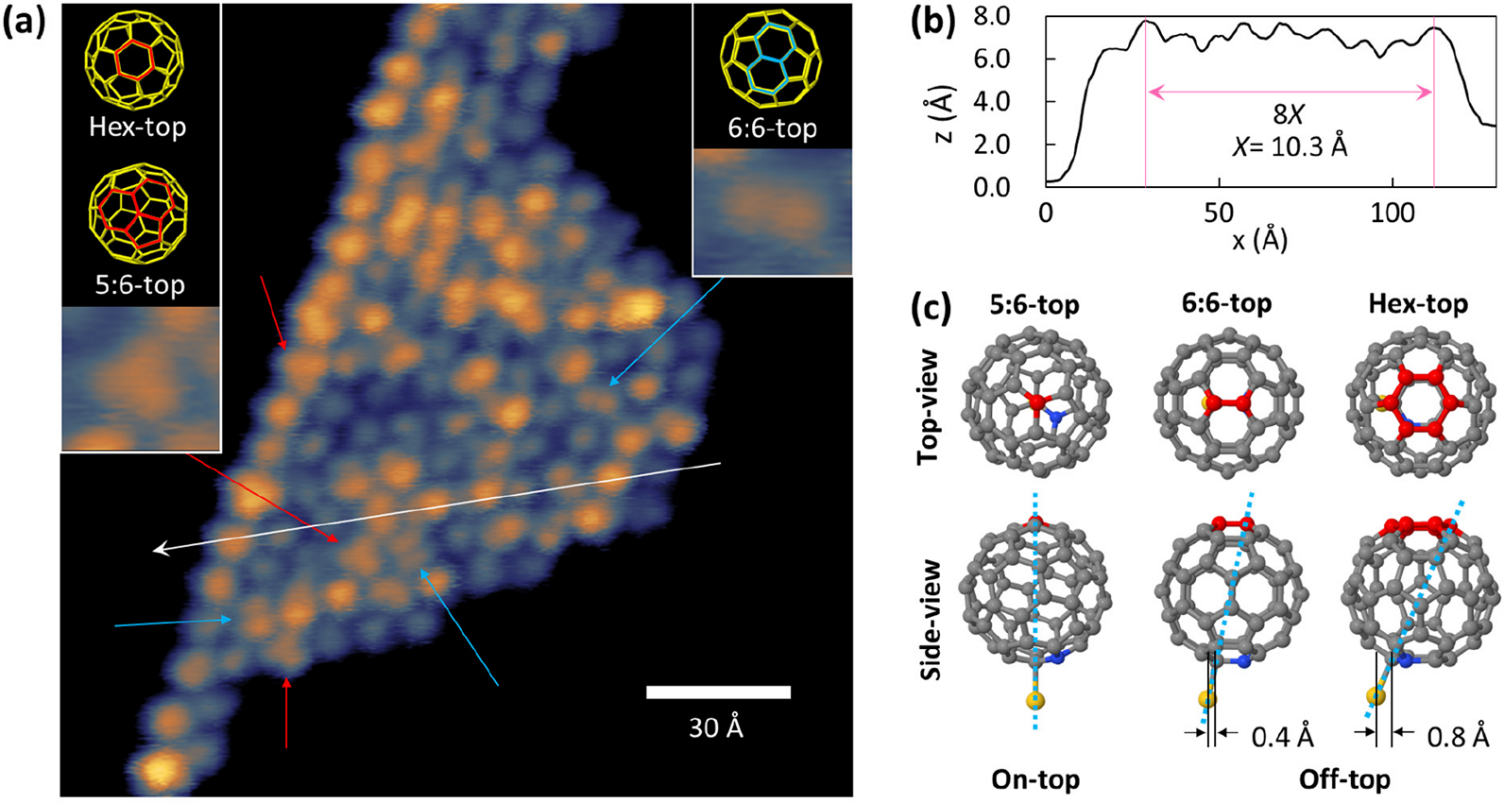
(a) STM image of C_59_N island formed on Au(111) substrate taken by a 100 pA tunneling current and 500 mV bias voltage. Red and blue arrows are pointing orbitals of 3-fold (5:6- or hex-top C_59_N, left inset) and 2-fold (6:6-top C_59_N, right inset) symmetries, respectively. White arrow shows the direction of [0-11] and is the cross-section used for the x–z analysis (b). (c) When C_59_N shifts away from the position exactly above the Au atom in the substrate layer (side-view), the top view of the molecule changes from the approximately 3-fold to 2-fold and back to 3-fold symmetry (top). Gray, red, blue, and yellow balls represent atoms of C, C at the top, N, and Au, respectively. Dashed cyan lines show the tilting of azafullerene.

Topographic STM images of deposited C_59_N material on the Au(111) surface reveal two-dimensional islands of hexagonally packed azafullerenes that start to grow at the step edges of the Au substrate (Figure 1a). These islands are orientationally commensurate with the Au(111) with close packed rows of molecules aligned with the rows of Au surface atoms running along [0-11], [-110], and [10-1], respectively. A line profile along [0-11] has a height difference of ∼6–8 Å with the substrate (Figure 1b) showing that the observed island of C_59_N is a monolayer. Measured average center-to-center ball distances along the three different principal directions are 10.2, 10.4, and 10.3 Å (Figure 1b, Figure.S1b), respectively. These distances are considerably longer compared to the (C_59_N)_2_ intradimer center-to-center distance of 9.41 Å and are comparable to interdimer center-to-center distances of 9.81 and 10.11 Å in the (C_59_N)_2_ solid.^20^ This suggests that the interaction between the electron in the frontier molecular orbital with the Au surface and the π–π interaction between azafullerenes defines the hexagonal packing of the islands as well as their relative orientation.

The interaction of adsorbed C_59_N with the Au(111) most likely proceeds with the C_59_N^•^ nonbonded carbon orbital neighboring the nitrogen that is oriented toward the Au surface. Our NEXAFS experiments as well as DFT calculations presented below indeed confirm this. Interestingly, careful examination of low-temperature STM images suggest that C_59_N^•^ bonded to the substrate are not uniformly adsorbed at the same orientation, nor at a uniform height from Au surface (insets to Figure 1a). The difference in the Au–C contact angle measured against the normal to the Au surface (Figure 1c) results in at least two different orbital shapes, holding 3-fold and 2-fold symmetry, when observed from the top. The orbital shape with a 2-fold symmetry corresponds to the C_59_N orientation with the hexagon–hexagon bond at the top (6:6-top, inset to Figure 1a). On the other hand, the 3-fold symmetry of the molecular orbital is approximately reproduced in cases when the azafullerene slightly tilts bringing the vortex between the two hexagons and the pentagon to the apex (5:6-top) or when the displacement of the azafullerene is even larger bringing the hexagon to the top (hex-top, inset to Figure 1a and Figure 1c). We stress that some tilting of the C_59_N monomers is necessary in order to accommodate molecules adsorbed to the Au(111) lattice. Similar orbital shape patterns were previously observed also for the C_60_ monolayer films on an Au(111) substrate.^21^

DFT calculations of a C_59_N monolayer on Au(111) confirm that there is an energetic preference for the azafullerene to orient with the carbon dangling bond toward an Au-atom in the layer below, at an Au–C distance of 2.22 Å (Figure S2). This puts the nitrogen atom at a 27° angle from the surface normal. Rotating the C_59_N about its center of mass in a series of single-point energy calculations shows the binding rapidly drops with angle, reaching >0.5 eV less stable for rotations above 15° (Figure S2). This suggests the monomers will only be surface oriented with relatively small angular variation, in agreement with the STM observation.

In contrast to the case of C_59_N adsorbed on Cu(111),^17^ Mulliken population analysis suggests weak charge transfer to the substrate (∼0.10*e* per cage). However, projected density of states (Figure S3) shows the azafullerene states near the Fermi level are strongly dispersed, indicating strong mixing with gold and as such Mulliken estimates are likely to be inaccurate.

We next calculated C_59_N at 25% surface coverage (4 × 4 Au supercell). A comparison to isolated gas-phase C_59_N gives a binding enthalpy of 1.216 eV per C_59_N to pristine flat Au(111). This rises to 2.837 eV when the C_59_N is in the 100% monolayer, clearly showing the driving force for close packing as seen in STM. Repeating these calculations without Au present shows that 0.567 eV of this increase in binding comes from interaction between neighboring fullerenes in the monolayer. This does not, however, account for all of the binding increase calculated upon azafullerene close-packing. This suggests therefore that close packing must also increase the Au–C_59_N interaction. Adding a second monolayer of C_59_N^•^, the binding energy per C_59_N^•^ in this layer decreases to 1.392 eV, around the half in comparison with the first monolayer, confirming a strong thermodynamic drive to form complete monolayer surface coverage of Au before C_59_N will deposit on top. This is also consistent with the STM data where close-packed surface monolayers form in preference to three-dimensional island clusters and corroborates again the strong Au–C_59_N^•^ interaction.

**Figure 2:**
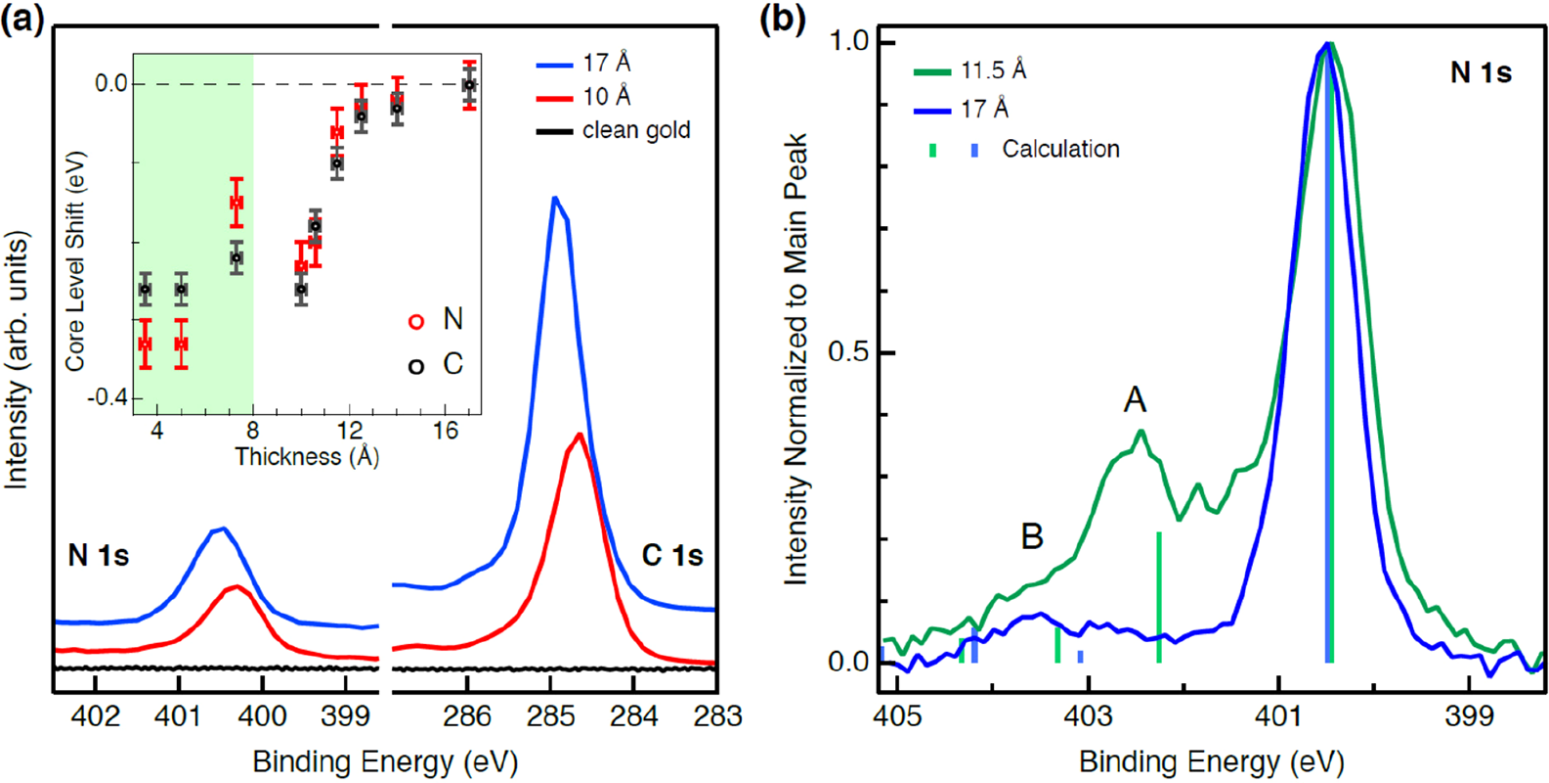
(a) XPS of C 1s, N 1s measured for 10 Å deposited C_59_N film (∼1.2 ML red curve) and for 17 Å film (∼2.1 ML, blue curve). Inset: the C 1s and N 1s screening shift with respect to Au(111) for increasing coverage. Crossover from 1st to 2nd layers is indicated at 8 Å by the green-white shading boundary. (b) XPS N 1s shakeup spectrum for 11.5 Å (green line) and 17 Å (blue line) thick layers of C_59_N/Au(111). Theoretically calculated peaks for the (C_59_N)_2_ dimer and C_59_N^•^ monomer taken from ref (23) are marked with green and blue vertical lines, respectively. The spectrum of the 11.5 Å thick layer (green) shows a prominent satellite peak at 402.3 eV (feature A) and a smaller satellite peak at 403.1 eV (feature B) that qualitatively match with the calculated satellite peaks for the radical monomer, whereas the spectrum of 17 Å (blue) displays two peaks at 402.7 and 403.5 eV that match closely the calculated satellite peaks for the (C_59_N)_2_ dimer.

The STM and DFT results demonstrate considerable interaction between the adsorbed C_59_N^•^ and the Au(111) surface within the first monolayer, but do not provide a clear insight into the radical state of the C_59_N entities. We thus turn to X-ray spectroscopy. In Figure 2a we compare the C 1s and N 1s photoemission peaks for 10 and 17 Å thickness azafullerene films on Au(111). For the 17 Å film thickness, the 1s core levels of C and N are observed at binding energies of 285.0 and 400.6 eV, respectively, in agreement with the values for thick (C_59_N)_2_ films reported in the literature.^13,22^ Relative to these two reference binding energies, the C 1s and N 1s peaks are systematically shifted to lower binding energies by about 0.25 and 0.35 eV for film thicknesses up to 8 Å (inset to Figure 2a), respectively. This energy shift is attributed to core-hole screening by the Au(111) surface and predominantly applies to the molecules closest to the Au surface, i.e., molecules within the first monolayer. The change of binding energy observed beyond 8 Å can be associated with the crossover from the first to second layer, where core-hole screening by the Au substrate is suppressed. The relative sharpness of the binding energy change indicates that C_59_N initially uniformly covers the Au(111) surface up to 1 ML (8 Å) before the second layer growth sets-in, just as observed by STM (Figure 1a) and DFT calculations. We note that the N 1s XPS peak displays a constant peak profile throughout the 0–8 Å coverage range without significant broadening, indicating that the distribution of C_59_N–Au contacts remains nearly the same throughout the monolayer coverage range. In fact, any significant reorientation of N within the fullerene cage in the first layer would introduce a different spread of binding energy shifts due to Au screening, which is not observed. We also performed cutoff photoemission measurement to determine work function for the submonolayer (3.5 Å) shown in Figure S4. We finally note that XPS spectra do not change within the experimental time of several hours, implying that C_59_N films are very stable under inert conditions for all coverage thicknesses.

The electronic nature of the adsorbed C_59_N^•^ may be further understood from analysis of the shakeup satellites of the C 1s and N 1s XPS peaks. In the C 1s shakeup spectrum of C_59_N^•^ films on Au(111) we observe a shoulder in the high energy tail of the C 1s peak at around ∼1 eV from the main elastic line (Figure S5). The energy difference between the elastic line and the shakeup satellite (photoelectron energy loss) corresponds to electron excitation from the occupied to unoccupied states (e.g., HOMO to LUMO) and has been previously observed in bulk films of (C_59_N)_2_ but not for C_60_ films. Therefore, it can be related to the sp^3^ carbon next to N in the azafullerene cage, which in monolayers bonds to the Au and in multilayer films cross-links to form a dimer.^22^

However, according to the calculated C 1s shakeup excitations for different C_59_N derivatives,^23,24^ the C 1s shakeup peaks are not sensitive to the radical state of the azafullerene films (Figure S5). We thus rather turn to the shakeup satellites of the N 1s XPS peak (Figure 2b) where we observe a noticeable difference between the films of 11.5 and 17 Å film thickness (∼1.4 and ∼2.1 ML, respectively). For the ∼2.1 ML film a faint peak (A) shifted by ∼2.2 eV and a second peak (B) by ∼3.1 eV from the main elastic peak are measured. On the other hand, the shakeup spectrum for 1.4 ML sample shows a pronounced peak centered at ∼1.8 eV from the N 1s main line. The nature of these loss peaks can be explored with the help of theoretical calculations for the C_59_N^•^ monomer and (C_59_N)_2_ dimer structures.^23^ The calculated positions of A and B peaks and the overall shape of the loss structure for the (C_59_N)_2_ dimer agrees well with our spectrum for the film-thickness of 17 Å, confirming that C_59_N^•^ forms dimers at 2.1 ML. This is also in agreement with the previous report for the azafullerene multilayer films.^13,14^ In contrast, the N 1s shakeup spectrum of the 11.5 Å film closely resembles the calculated shakeup structure for the C_59_N^•^ monomers in their radical state. In particular, the loss peak at ∼402.3 eV binding energy, which matches the calculated position and relative intensity, is exclusively predicted for the C_59_N^•^ monomer, but not for other candidate species like (C_59_N)_2_ dimers. Both the shift and the relative increase in intensity of this peak are consistent with the radical character of C_59_N^•^ monomers in the monolayer. Namely, the radical orbital, although primarily centered on the C site neighboring N, exhibits significant overlap with the nitrogen.^23^ This overlap is for example also evidenced by the characteristic hyperfine splitting in the EPR spectra of C_59_N^•^ radicals.^7−9^ So, we conclude that the measured shakeup structure of C_59_N^•^ monomers reflects their radical state (Figure 2b).

**Figure 3:**
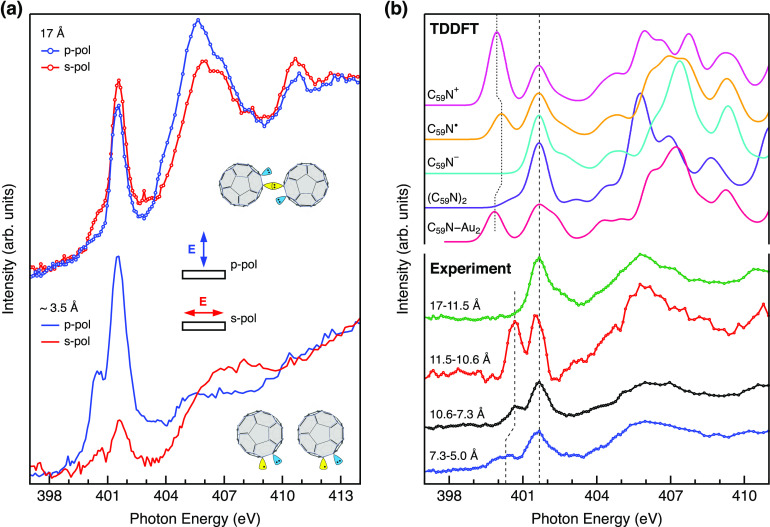
(a) N K-edge NEXAFS linear dichroism for 3.5 Å thick film C_59_N (lower panel) and a 17 Å thick film (upper panel). The average orientation of the N site of the azafullerene cage is indicated in the insets. (b) Layer resolved sequence of differential NEXAFS in “magic” superposition of p-polarization and s-polarization spectra are compared with the calculated ones for the (C_59_N)_2_, C_59_N^–^, C_59_N^•^, and C_59_N^+^ molecules and the artificial C_59_N–Au_2_ complex with 2.0 Å coupling distance, respectively. For the calculated spectra, we used time-dependent DFT (TDDFT) calculations (see Methods for details). All spectra are intensity scaled to the probed coverage, and offset vertically for clarity.

We now turn to the NEXAFS spectra. Both C 1s and N 1s NEXAFS for the 17 Å (∼2.1 ML) C_59_N film (Figure 3a, Figure S6, and Figure S7a) closely resemble those reported earlier for (C_59_N)_2_ thick films.^13,22^ The principal peaks are due to the transitions from the core level to the LUMO and above. We note that the C 1s → LUMO (LUMO+1) resonance at the photon energy *h*ν = 284.9 eV (286 eV) aligns with the N 1s → LUMO (LUMO+1) resonance at 400.7 eV (401.6 eV), see Figure S7b. The same alignment has been previously reported also for (C_59_N)_2_ bulk films.^13,22^ The N 1s → LUMO peak at 400.7 eV shows only as a shoulder, indicating a weak LUMO resonance with the N 1s core level (Figure 3).

Next, we compare the N 1s NEXAFS spectra for 3.5 Å (0.4 ML) and 17 Å (2.1 ML) films. The spectra shown in Figure 3a were taken with the photon polarization along the surface normal (p-polarization) and parallel to the surface (s-polarization). The pronounced linear dichroism of the main absorption lines (π* symmetry resonances) at 3.5 Å indicates that molecules of the first layer adopt a rather uniform adsorption configuration with N site of the azafullerene cage oriented toward the substrate at 30 ± 3° average angle from the surface (inset to Figure 3a). This is in excellent agreement with the low-temperature STM experiments and DFT computations discussed above (Figure 1 and Figure S2) and experimentally demonstrates that C_59_N^•^ indeed couples to Au(111) via the cage orbitals on carbon next to the substitutional nitrogen atom.

Interestingly, the N 1s NEXAFS spectra taken with the same photon polarizations on 17 Å film with two completed layers of C_59_N^•^ lack this strong dichroism indicating that the second layer adopts very different geometry (Figure 3a). Instead, the intensity of these N π* resonances in s-polarization even exceeds that of p-polarization, pointing to an average orientation of nitrogen atoms in the completed second layer close to 60° from the surface. This agrees well with (C_59_N)_2_ dimer formation of the second layer molecules with the C–C bond oriented in-plane and thus with N orbitals oriented predominantly at ∼60° from the surface.

In order to follow the evolution from the radical monolayer to the second layer of (C_59_N)_2_ dimers, we next performed differential N 1s NEXAFS analysis by sequentially subtracting spectra taken with increasing coverage (Figure 3b). In total, five films in the thickness range between 5 and 17 Å have been investigated. All spectra have been taken in two orthogonal polarizations (s- and p-polarizations) and then added by a linear combination (*p + 2s*) into a synthetic spectrum corresponding to the magic angle. Such “magic” spectral representation has the advantage of being independent of molecular orientation, which as we have just argued, changes with coverage, and therefore reflects only the orbital structure of the added film. Figure 3b shows the resulting sequence of four “magic” NEXAFS spectra resolved in thickness: (7.3–5 Å), (10.5–7.3 Å), (11.5–10.5 Å), and (17–11.5 Å), respectively. The spectra show a strong variation especially in the low energy absorption line at ∼400.5 eV. This peak displays the largest intensity for the supramonolayer coverage (11.5–10.5 Å), whereas it is completely quenched for the coverage (17–11.5 Å) when the coverage approaches the completion of the second layer. The submonolayer film (7.3–5 Å) displays only moderate intensity of the 400.5 eV peak and even a small shift (∼0.3 eV) to lower energies.

The observed evolution of differential N 1s NEXAFS spectra can be corroborated with DFT calculations of N 1s NEXAFS spectra for the (C_59_N)_2_ dimer and neutral and charged C_59_N monomers (Figure 3b). Calculated N 1s NEXAFS spectrum for the (C_59_N)_2_ dimer yields excellent agreement with the experimental (17 Å-11.5 Å) spectrum, confirming dimer formation at the completion of the second layer. A close inspection of DFT results reveals that the LUMO of (C_59_N)_2_ is mainly delocalized across the carbon sites with minimal overlap with the N sites.^22^ This agrees with the observed low intensity of the N 1s → LUMO peak at 400.7 eV measured in the ∼2 ML films. Considering instead the C_59_N^•^ monomer, our DFT calculations show that the LUMO of the monomer now spreads also over N (see also ref (10)). This indicates that the LUMO orbital of the dimer turns into SUMO for the monomer, which also agrees with the observed intensity increase of the lowest energy peak at 400.7 eV in NEXAFS for the monomer films.

When adsorbed on Au, the C_59_N^•^ monomer will align its SUMO/SOMO (SOMO = singly occupied molecular orbital) energy to the Fermi level, enabling charge transfer with the substrate. From our DFT calculations, we observe that the dangling radical is a half-filled electronic state at the Fermi level. Removing further charge empties this state and causes a downshift in its energy. In contrast, adding a second electron completely fills the state and hence quenches its signal in NEXAFS (Figure 3b). The N 1s NEXAFS peak at ∼400–401 eV can therefore be attributed to the N 1s excitation to the LUMO/SUMO level, depending on the occupancy of this level, i.e., the charge state. The energy position and relative intensity of the N 1s NEXAFS peak at ∼400–401 eV are therefore very sensitive probes of the LUMO/SUMO orbital population of the C_59_N radical state. To corroborate this finding, we finally computed NEXAFS spectra for artificial C_59_N–Au_2_ complexes to emulate the interaction between adsorbed C_59_N^•^ and the Au substrate. Computations were performed for different C–Au bond lengths ranging from 2 to 4 Å (Figure S8). We report here results with the Au–Au distance fixed at 2.6 Å. For the largest C–Au distance of 4 Å, the C_59_N–Au_2_ interaction is weak and thus its N 1s NEXAFS spectrum resembles that of the C_59_N^•^ in the gas phase. On the other hand, as soon as carbon of C_59_N approaches Au at distances smaller than 3 Å, we notice a systematic shift of the SUMO peak to lower energies. Therefore, increasing the interaction between C_59_N^•^ and Au results in the increase in SUMO-LUMO splitting. These additional calculations thus finally explain the fine variations in the differential N 1s NEXAS spectra. For the (7.3–5.0 Å) spectrum the first layer of C_59_N^•^ interacts with the Au surface and thus the SUMO splitting is larger than for the (11.5–10.5 Å) spectrum where the second layer of C_59_N^•^ is already distanced from the Au surface and thus the interaction with the substrate is negligible.

**Figure 4:**
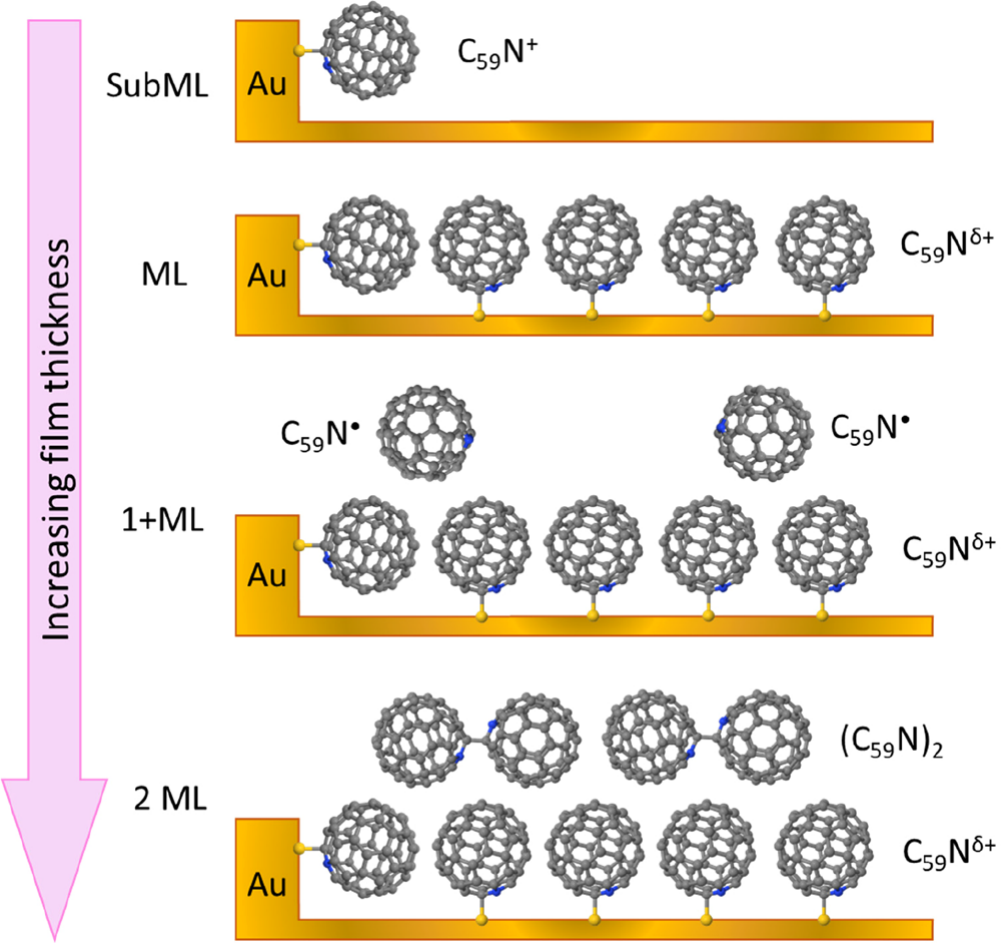
Evolution of C_59_N^•^ radical character as a function of the azafullerene film thickness on Au(111). Gray, blue, and yellow spheres represent C, N, and Au atoms, respectively.

We can now build a complete picture of C_59_N^•^ radical state evolution as the thickness of the film on the Au(111) surface increases (Figure 4). At very low C_59_N coverage, e.g. (7.3–5 Å), the differential N 1s NEXAFS spectrum shows a broad SUMO peak around ∼400 eV indicating different charge states of the adsorbed species. This is due to partial quenching of the radical character of selected C_59_N^•^ monomers, induced by site dependent C_59_N–Au coupling and possibly accompanied by a partial charge transfer (see section SA in Supporting Information for an extended discussion of the C_59_N radical state within the first monolayer). The coupling to the surface may be particularly favored at Au sites with reduced coordination, such as step edges, where molecules preferentially adsorb, as observed by the STM.

Addition of C_59_N^•^ monomers promotes the growth of the hexagonally packed islands of C_59_N^•^ monomers that orient their reactive carbons next to N predominantly toward the Au. Due to their incommensurability with the Au(111) surface, azafullerene islands display significant variations in the Au–C_59_N contact distance. We stress that the Au lattice mismatch imposes some tilting and lifting of fullerene cages, directly seen in our STM images (Figure 1c). According to the DFT calculations, such local contact variations tune the charge transfer between the substrate and C_59_N (section SA in the Supporting Information), yet they keep the nitrogen of the azafullerene cages oriented toward Au at ∼30° from the (111) normal. This interacting contact layer of C_59_N monomers then serves as a buffer layer and a template for the growth of the subsequent second layer of C_59_N^•^ radicals. In this second layer, the coupling to the substrate is negligible as concluded from (*i*) both C 1s and N 1s XPS binding energies reproduce those of a multilayer system and (*ii*) the lack of linear dichroism in N 1s NEXAFS spectra, which indicates that C_59_N are no longer orientationally locked and can now orient freely in space. Provided that the C_59_N filling of the second layer is low as is the case for (10.5–7.3 Å) and (11.5 Å-10.5 Å), these second layer molecules remain mostly isolated monomers with highly expressed radical character. They are therefore recognized as an intriguing high spin-density phase. Upon further increase of the second layer filling (approaching 2 ML as is the case for (17–11.5 Å)) statistically, more and more azafullerenes in the second layer find themselves in next neighbor positions, where they are able to reorient and couple in (C_59_N)_2_ dimers. Upon dimerization, they lose their radical character, and the N 1s NEXAFS resonance at 400.5 eV disappears. The dimerization into nonmagnetic (C_59_N)_2_ therefore starts as early as the second layer approaches full completion.

## Conclusions

In conclusion, we explored the sensitivity and high resolution of low-temperature STM and the XPS/NEXAFS spectroscopy supported by DFT computations to study the stability of C_59_N^•^ radicals on the Au(111) surface when deposited in a vacuum by thermal sublimation. In the first monolayer of C_59_N, adsorbed azafullerenes form a hexagonal lattice of monomers, where they retain their monomer character but on average partially lose their radical character due to interaction with Au. Although the particular molecular orientation where the azafullerene carbon dangling bond directly faces a Au atom while at the same time the nitrogen orients toward the substrate at 30 ± 3° average angle remains robust over the first monolayer, local variations in the Au–C_59_N distance result in the variations in the charge transfer between C_59_N and the substrate. Such a first contact monolayer with nonuniform quenching of the radical state nevertheless serves as a passivating template layer for additional azafullerenes that are deposited on top. Isolated orientationally disordered C_59_N^•^ monomers in the second layer are uncoupled and fully retain their radical state. The sacrificial role of the first layer can then be exploited to form stable radical monomers in the second, noncontact layer. In-plane dimerization of the second layer molecules begins only for higher coverages, on approaching the completion of the second layer.

The discovery of a supramonolayer phase of azafullerene monomers with expressed radical character on top of a passivating C_59_N monomer layer/Au(111) therefore demonstrates that spin active azafullerene radicals may be formed when weakly coupled to the substrate. Due to the extremely long coherence times of C_59_N^•^ spin states,^10^ the possibility of having stable two-dimensional C_59_N^•^ lattices may provide opportunities for the coherent manipulation of molecular spin qubits. Moreover, the robustness of C_59_N^•^ radical states for film thicknesses is important for the design of surface fullerene synthetic and catalytic processes. This simple approach of first forming a stable nonradical sacrificial layer before subsequently depositing spin active molecules may also be applicable for other molecular radical systems.

## Methods

### (C_59_N)_2_ Synthesis and Characterization

Powder samples of azafullerene dimer (C_59_N)_2_ were synthesized following the standard procedure described in the literature.^5^ The sample purity was followed with high-performance liquid chromatography (HPLC) where the HPLC trace after recycling shows a single peak (Figure S9). Next, (C_59_N)_2_ samples were characterized with ^13^C nuclear magnetic resonance (NMR) in deuterated ortho-dichlorobenzene. The ^13^C NMR spectrum (Figure S10) shows a number of sharp peaks in the range between 136.4 and 148.7 ppm and separated peaks at 155.8 and 125.1 ppm, all of them due to *sp*^2^ carbons. The separated peak at 108.0 ppm is due to the interazafullerene bonding carbon sites with carbon orbitals close to sp^3^. All these peaks are fingerprints of (C_59_N)_2_ and are in full agreement with the data reported in the literature^25^ and at the same time confirm high purity of our samples.

### Low-Temperature Scanning Tunneling Microscopy

Prior to deposition and low-temperature STM measurements, special care was taken to thoroughly clean the Au(111) surface and azafullerene sample material. The Au(111) surface was cleaned using the standard Ar^+^ sputtering (10 μA, 1 kV) and annealing (up to 800 K) cycles until a clean and well-ordered surface showing Herringbone reconstruction was obtained. The azafullerene molecules were deposited from a (C_59_N)_2_ powder by using a homemade evaporator, consisting of a quartz tube with a tungsten filament wrapped around it. Due to the design of the evaporator, it was not possible to reliably measure its temperature during deposition. Thus, to ensure good reproducibility, the heating current was used as a measure of the evaporation rate. First, the molecules were degassed for several days at 1.6 A in UHV. For each deposition, the current was ramped over the course of several hours to 4.0 A, keeping the pressure in the evaporation chamber in the 10^–10^ mbar range.

After the deposition, the samples were immediately transferred to the Specs low-temperature JT-STM with a base temperature of 4.2 K. Measurements were performed using electrochemically etched W tips, reconditioned in situ by controlled tip–sample interaction, and characterized on a clean Au(111) or Ag(111) surface. Topography images were taken in constant current mode (*I* = 10–200 pA). At negative bias voltages electrons dominantly tunnel from the sample into the tip, allowing us to image occupied states while using positive bias voltages to probe the unoccupied states of the sample. In order to precisely determine intermolecular distances, the microscope calibration was verified using known herringbone reconstruction parameters.

### XPS and NEXAFS Spectroscopy

The XPS and NEXAFS measurements were performed at the ALOISA beamline of the Elettra Synchrotron Facility, Trieste.^26^ The Au(111) substrate surface was cleaned by repeated cycles of sputtering (1.5 kV Ar^+^) and annealing up to 723 K. The (C_59_N)_2_ was sublimed from a homemade boron nitride crucible, at a starting temperature of 670 K, up to 830 K; the increase in the sublimation temperature in order to deposit the same amount of material is possibly due to C_59_N polymerization reactions inside the crucible. The overall film thickness of the deposited C_59_N has been determined from the intensity attenuation of the Au 4f peak due to inelastic scattering of photoelectrons passing through the organic overlayer.^27^ Core level photoemission data were acquired in normal emission geometry with a constant 4° grazing angle of a linearly p-polarized light beam with respect to the Au(111) surface plane. The C 1s and N 1s XPS spectra were acquired at a photon energy of 515 eV with a total energy resolution of 160 meV. Binding energies were calibrated with respect to the bulk spectral component of the Au 4f_7/2_ peak at 84.0 eV.^28^ C–K edge and N–K edge NEXAFS spectra were acquired in partial electron yield mode by means of a channeltron multiplier equipped with a negatively biased grid to filter out low energy secondary electrons in order to improve the signal to background ratio. In order to investigate the sample linear dichroism, the NEXAFS spectra were acquired at two different orientations of the synchrotron beam with respect to the surface plane, namely, transverse magnetic (p-polarization) and transverse electric (s-polarization) geometry, by sample rotation around the photon beam axis. The beam grazing angle was kept constant at 6°. The photon energy calibration and photon flux normalization methods are described in detail elsewhere.^29^ Both XPS and NEXAFS measurements were carried out with the sample temperatures between 300 and 430 K.

### DFT Calculations

Ground-state density functional (DFT) calculations were carried out using the AIMPRO code^30−32^ using the local density approximation for exchange-correlation energy.^33^ Kohn–Sham wave functions for C and N are constructed using localized Gaussian orbital functions multiplied by polynomials, with 38 and 40 independent functions, respectively (*l* ≤ 2), with plane-wave energy cutoff of 175 Ha. Calculations were spin optimized and polarized with different integer spin states tested (only the most stable are reported here). Relativistic pseudopotentials given by Hartwigsen, Goedecker, and Hutter (HGH)^34^ with a finite electron Fermi temperature of kT = 0.001 eV were utilized. Systems were geometrically converged within 10^–5^*a*_B_ in position (here *a*_B_ is Bohr radius) and 10^–6^ Ha in energy. To investigate angle-energy dependency of the adsorbed C_59_N, single point energy calculations were carried out changing only the angle of the C_59_N molecule with respect to its center of mass, without letting any atoms relax (i.e., optimize their positions). This constraint is imposed in order to avoid unintended cage movement.

For the hybrid system calculations, 2 × 2 supercell Au_36_ triple-layer Au(111) slabs were created in hexagonal supercells, allowing atoms and lattice parameter to vary with a 6 × 6 × 1 k-point mesh, until reaching convergence (a_0_ = 9.72 Å). A cell *c*-axis spacing of >26 Å is chosen and fixed, to ensure there is no interaction between C_59_N^•^ and the base of the neighboring slab. The C_59_N spacing is essentially imposed by the choice of supercell and as such should not be compared directly to the experimental STM spacing. A single C_59_N was added in close-packed direction oriented along [1–10] on the unreconstructed Au(111) trilayer (9.72 Å C_59_N spacing, i.e. 1 C_59_N per 2 × 2 Au supercell), all atoms allowed to fully relax, with lattice parameters fixed. Once converged, the C_59_N was then rotated about its center of mass in steps of 5°/10°, and single point energy calculations were performed to explore the energy barrier for molecular rotation. Azafullerene charge state was determined through Mulliken population analysis.

To model the 2 ML system, a radical C_59_N^•^ was added on top of the first monolayer with the radical facing toward the Au layer. We assume AB azafullerene stacking and a cell *c*-axis spacing increase to >32 Å. All atoms were allowed to relax. To explore the effects of coverage density, a 144 atoms 4 × 4 Au supercell was created, where a single C_59_N^•^ was added on it (25% coverage), as well as (C_59_N)_2_ for a 50% coverage. All atoms were again relaxed with a 3 × 3 × 1 k-point mesh.

NEXAFS spectra for C_59_N^+^, C_59_N^•^, C_59_N^–^, and (C_59_N)_2_ were calculated using the ORCA code^35,36^ within a hybrid time-dependent DFT framework (TD-DFT). The hybrid TDDFT calculations were carried out at the B3LYP^37^/ZORA-def2-TZVP^38−40^ level with def2/J auxiliary basis set, spin-unrestricted SCF and RIJCOSX approximation, after DFT geometry optimization and frequency calculations at the B3LYP/6-31G**^41−46^ level. The default setting was used for the other parameters. For the C_59_N–Au_2_ complex, the def2-TZVP basis set was instead used for NEXAFS calculations.

## Supporting Information Available

Figure S1: STM image of C_59_N island on Au(111) substrate and its cross-section analysis along 3 different directions. Figure S2: DFT calculations of close-packed C_59_N^•^ monomers on Au(111). Figure S3: Projected density of states (eV) for C_59_N^•^ on Au(111) at low surface density calculated by DFT. Figure S4: X-ray photoemission cutoff measurements of surface work function. Figure S5: C 1s shakeup spectrum of C_59_N films on Au(111). Figure S6: C and N 1s NEXAFS spectra for 2.1 ML measured at the magic angle. Figure S7: Polarization NEXAFS spectra of C 1s and N 1s at different layers of C_59_N^•^. Figure S8: Calculated N 1s NEXAFS spectra of artificial C_59_N–Au_2_ complex with different C–Au bond lengths. Figure S9: HPLC trace of (C_59_N)_2_ sample. Figure S10: Solution ^13^C NMR spectrum of as-prepared (C_59_N)_2_ sample. Section SA: C_59_N radical state within the 1st monolayer. Figure S11: N 1s NEXAFS spectra for increasing C_59_N coverage. Figure S12: N 1s NEXAFS spectra of C_59_N with different film thickness and after annealing. Figure S13: Calculated bonding orbital and charge density plots of C_59_N on Au substrate. Figure S14: Plot of calculated total system spin as a function of Au–C distance. (PDF)

## Abbreviations

ML: monolayer
XPS: X-ray photoemission spectroscopy
NEXAFS: X-ray absorption fine structure spectroscopy
STM: scanning tunneling microscopy
DFT: density functional theory
SUMO: singly unoccupied molecular orbital
UHV: ultrahigh vacuum
HOMO: highest occupied molecular orbital
LUMO: lowest unoccupied molecular orbital
TDDFT: time-dependent density functional theory
SOMO: singly occupied molecular orbital.
