# Supplementary material for: Noncontact Layer Stabilization of Azafullerene Radicals: Route toward High-Spin-Density Surfaces
Source: ACS Nano. 2023 Dec 12;17(24):25301–10. doi: 10.1021/acsnano.3c08717 (PMC10753892; doi:10.1021/acsnano.3c08717)
Supplement: Supplementary file 1 — nn3c08717_si_001.pdf [file nn3c08717_si_001.pdf]

Supporting Information

Non-contact layer stabilization of azafullerene radicals: route towards high-spin-density surfaces

Yuri Tanuma[1,2#§], Gregor Kladnik[3,4§], Luca Schio[4], Marion van Midden Mavrič[1], Bastien Anézo[5], Erik Zupanič[1], Gregor Bavdek[4,6], Ruben Canton-Vitoria[7], Luca Floreano[4], Nikos Tagmatarchis[7], Hermann A. Wegner[8,9], Alberto Morgante[4,10], Christopher P. Ewels[5], Dean Cvetko[1,3,4*], Denis Arčon[1,3*]*

[1] Jožef Stefan Institute, Jamova 39, SI-1000, Ljubljana, Slovenia.

[2] Center for Advanced Research of Energy and Materials (CAREM), Hokkaido University, Kita 13, Nishi 8, Kitaku, Sapporo 060-8628, Japan.

[3] Faculty of Mathematics and Physics, University of Ljubljana, Jadranska 19, SI-1000, Ljubljana, Slovenia.

[4] CNR-IOM, Istituto Officina dei Materiali, Basovizza Area Science Park, I-34149, Trieste, Italy.

[5] Institut des Matériaux de Nantes Jean Rouxel (IMN), UMR 6502 CNRS, Nantes University, 44322 Nantes, France.

[6] Faculty of Education, University of Ljubljana, Kardeljeva ploščad 16, SI-1000 Ljubljana

Slovenia.

[7] Theoretical and Physical Chemistry Institute, National Hellenic Research Foundation, 48 Vassileos Constantinou Avenue, Athens 11635, Greece.

[8] Institute of Organic Chemistry, Justus Liebig University Giessen, Heinrich-Buff-Ring 17, 35392 Giessen, Germany.

[9] Center for Materials research (ZfM/LaMa), Justus Liebig University Giessen, Heinrich-Buff-Ring 16, 35392 Giessen, Germany.

[10] Physics department, University of Trieste, Via Valerio 2, 34012, Trieste, Italy.

§ Y.T. and G.K. contributed equally to this work.

* Corresponding authors:

chris.ewels@cnrs-imn.fr

dean.cvetko@fmf.uni-lj.si

denis.arcon@ijs.si

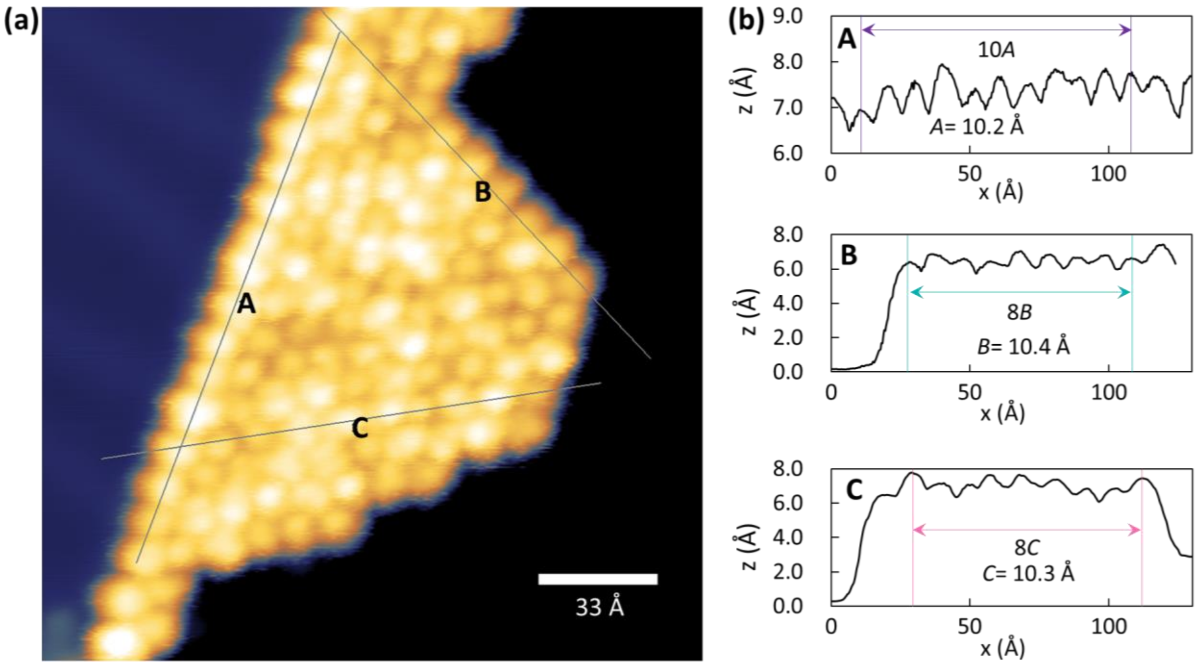

Figure S1. (a) STM image of $C_{59}N$ island on Au(111) substrate and (b) its cross-section analysis taken along the lines A, B and C in (a).

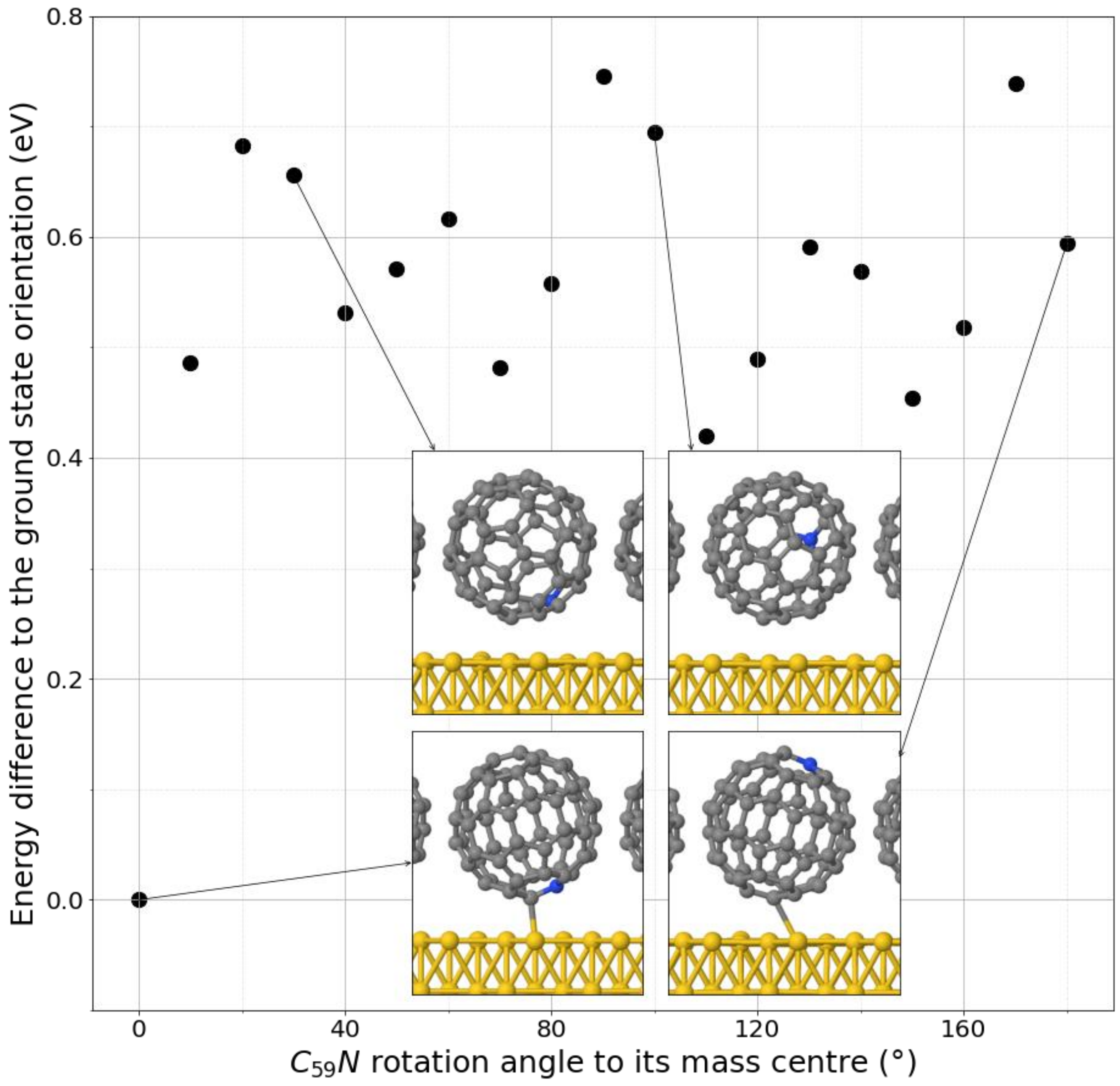

Figure S2. DFT calculations of close-packed $C_{59}N^{\bullet}$ monomers on Au(111). The system is fully optimized (fixed slab and allowed all atoms of $C_{59}N$ to vary) for the 0° case. Other points are generated by rotating $C_{59}N$ about its centre of mass from the 0° case without subsequent geometric relaxation. These energies confirm that there is an energetic preference for the azafullerene to orient with the carbon dangling bond towards an Au-atom in the layer below.

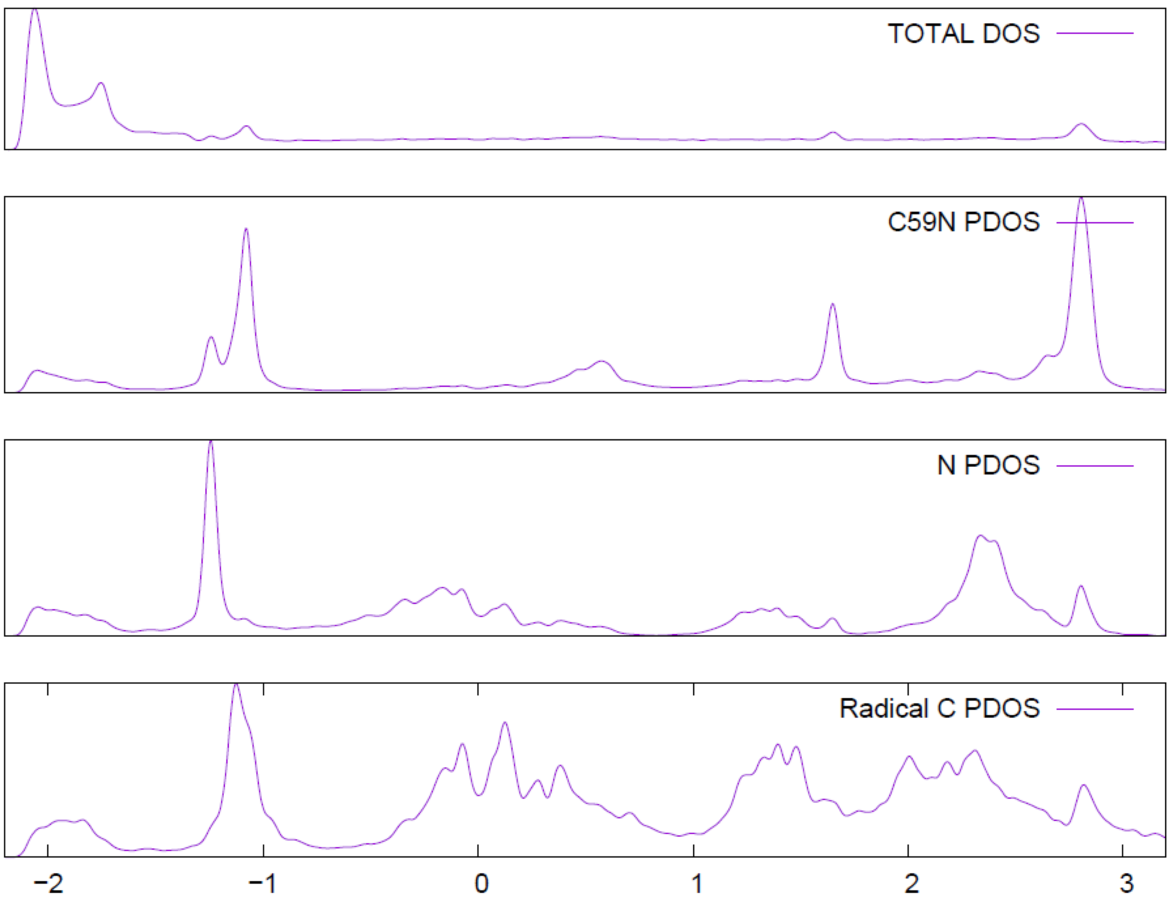

Figure S3: DFT Projected density of states (eV) for $C_{59}N^{\bullet}$ on Au (111) at low surface density. Each spectrum normalized to highest spectral peak (i.e., not constant normalization across all four spectra). Fermi level is aligned at 0 eV. The unbound carbon next to nitrogen ('radical C') shows highly dispersed levels around the Fermi level, indicating strong interaction with gold electronic states.

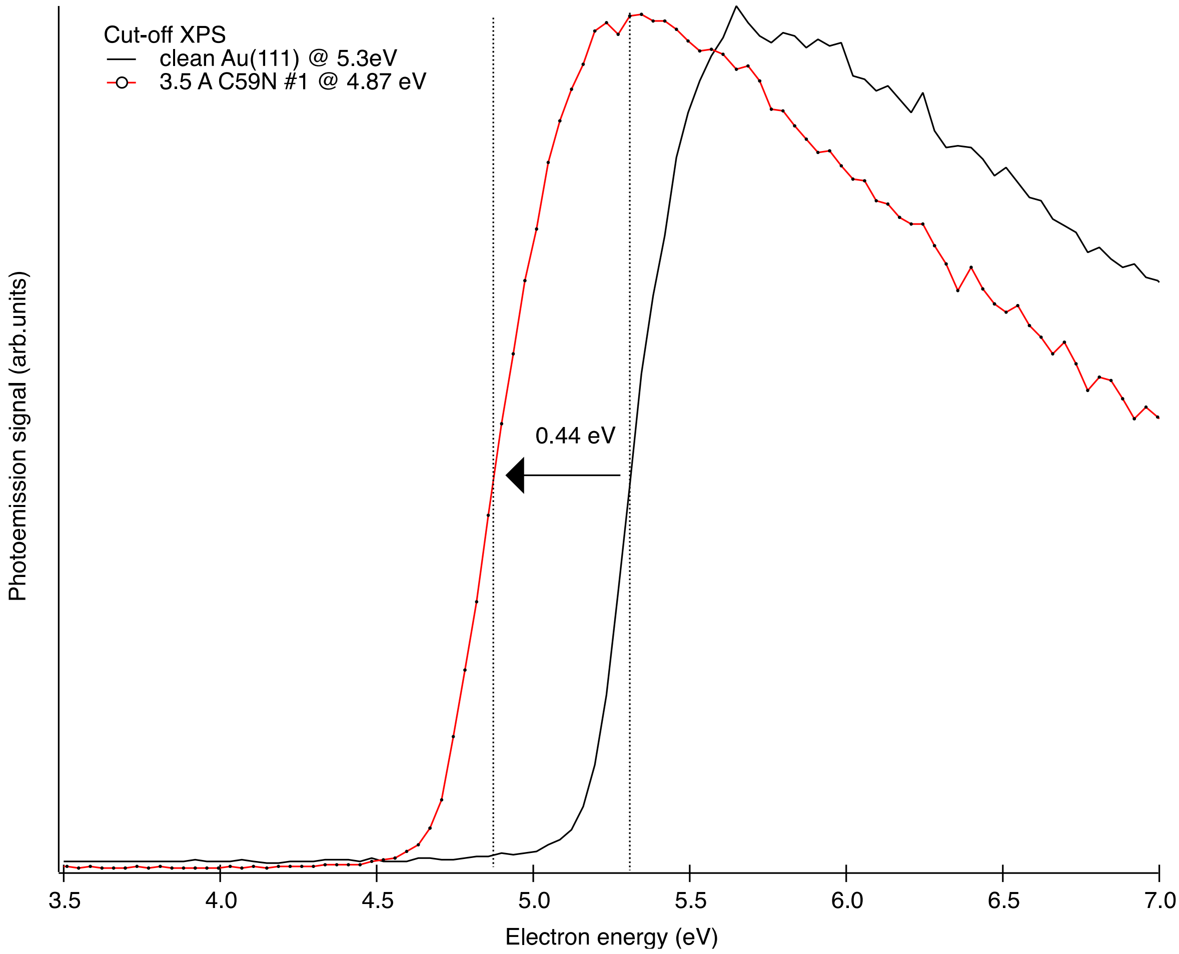

Figure S4. X-ray secondary electron cut-off measurements of the surface work function using low photon energy of ~150 eV. Bias voltage of -30.00 V was applied on the sample. X-ray photon energy was calibrated from the kinetic energy difference of the Au $4f_{7/2}$ photoemission peak (@ 84 eV B.E.) measured with the 1st and 2nd order lines of the ALOISA monochromator. The value of the surface work function is then obtained as a difference between the photon energy and the secondary electron emission bandwidth (low energy cut-off to Fermi edge). The work function reduction of 0.44 eV relative to the clean Au(111) may be observed for the 3.5 Å thick $C_{59}N$ layer, indicative of the organic layer interaction with the Au(111) surface.

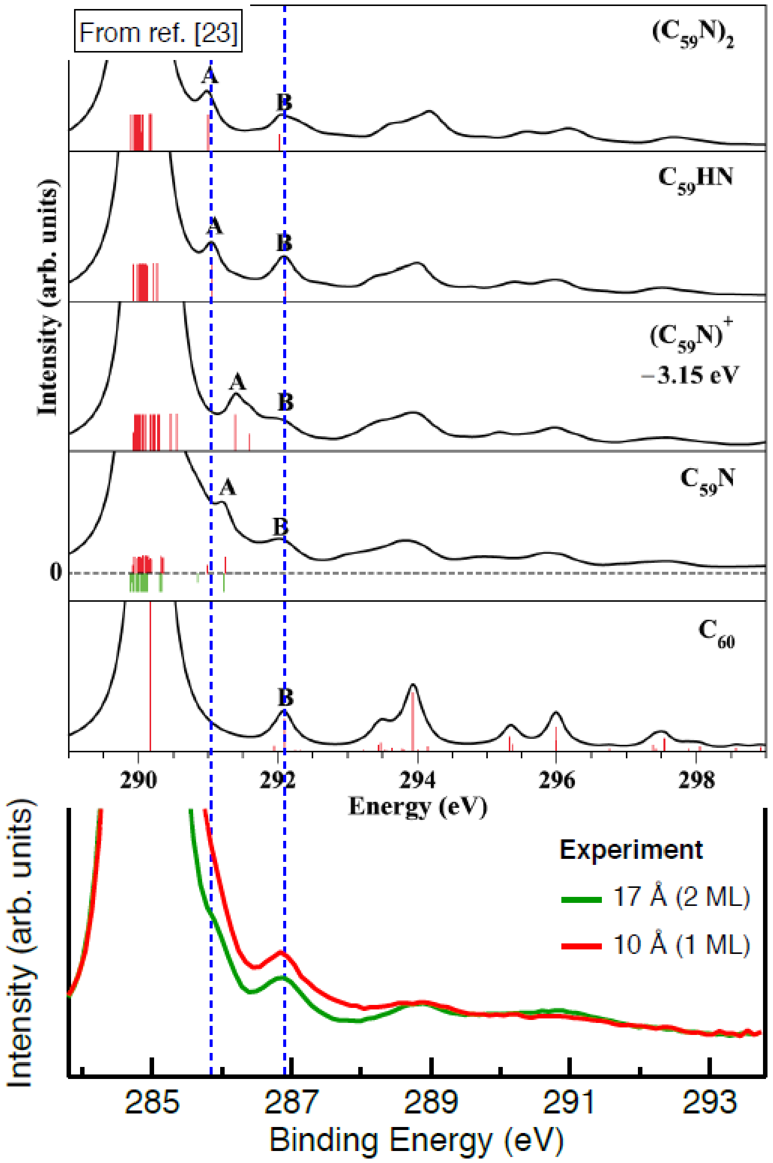

Figure S5. C 1s shake-up spectrum of $C_{59}N$ films on Au(111). Please note a shoulder in the high energy tail of the C 1s peak at around ~1 eV from the main elastic line. Comparison is included to theoretical shake-up spectra for different azafullerene species calculated and reproduced from Reference 23 (reprinted from [Deng, Y.; Gao, B.; Deng, M.; Luo, Y. A Comparative Theoretical Study on Core-Hole Excitation Spectra of Azafullerene and Its Derivatives. J. Chem. Phys. 2014, 140, 124304.], with the permission of AIP Publishing). Note that the calculated binding energies (upper panel) are referenced to the vacuum level, whereas the experimental binding energies (lower panel) are referenced to the Fermi level. Both spectra are aligned to the main peak of the photoemission line.

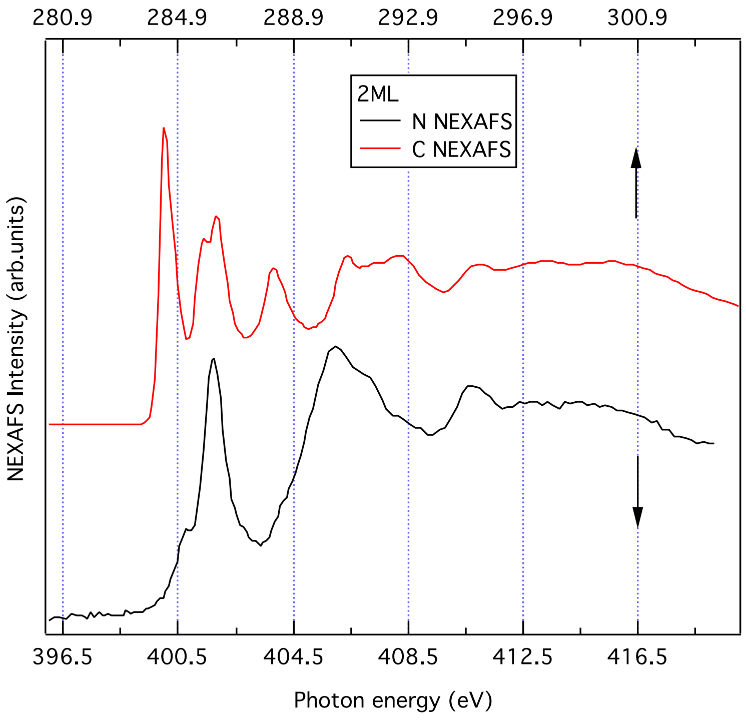

Figure S6. Carbon and nitrogen NEXAFS for the 2.1 ML film shown in the magic angle configuration. Upper and lower energy scales are aligned on the binding energies of respective XPS C 1s and N 1s peaks (284.9 eV and 400.5 eV). The photon energy scales for C (top axis) and N (bottom axis) are offset by 115.6 eV which is the binding energy difference between the respective 1s core levels.

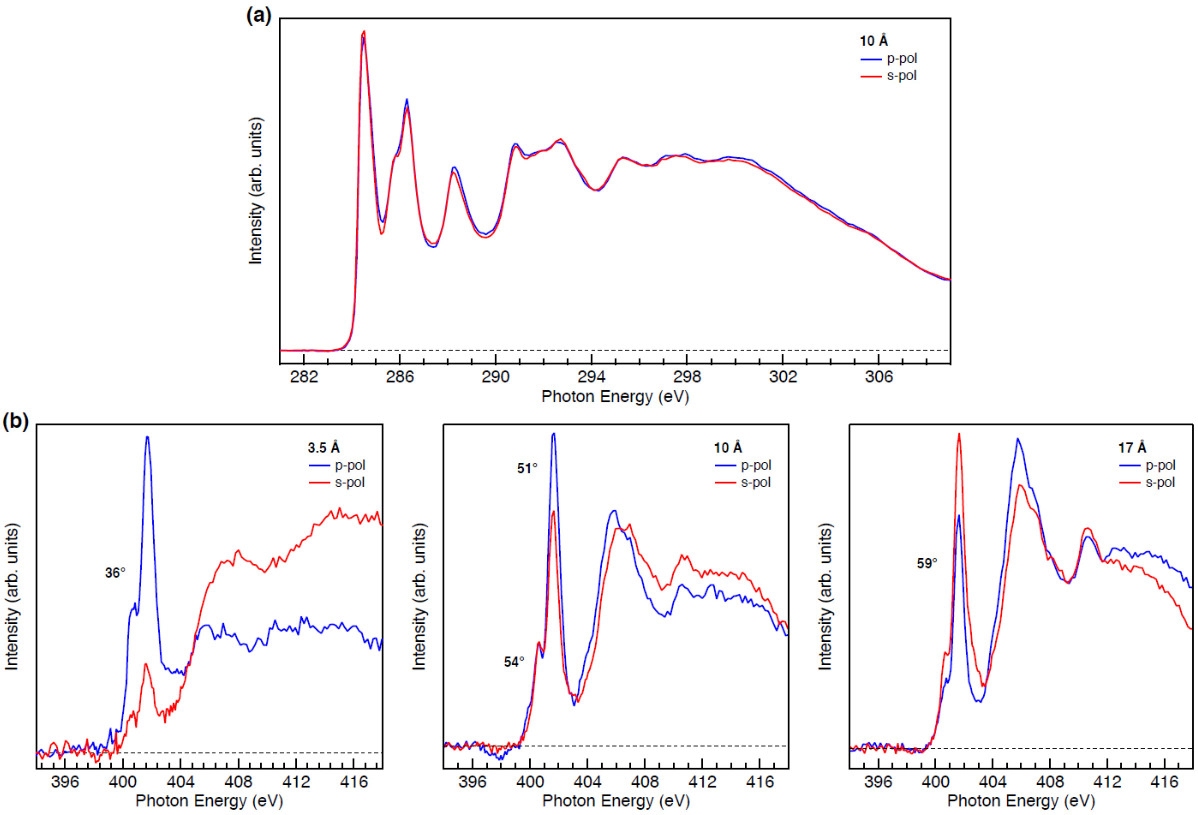

Figure S7. Polarization NEXAFS spectra of (a) C 1s and (b) N 1s at sub-ML (3.5 Å), 1 ML (10.6 Å), and 2 ML (17.0 Å) of $C_{59}N^{\bullet}$. Blue and red spectra were taken with the photon polarization along the surface normal (p-polarization) and parallel to the surface (s-polarization), respectively. From the intensity ratio of the π^* peak measured in p-pol and s-pol configurations we obtain the orientation (θ) of the N site within the fullerene cage from the surface. Calculated angle θ for (b) is shown in the figure.

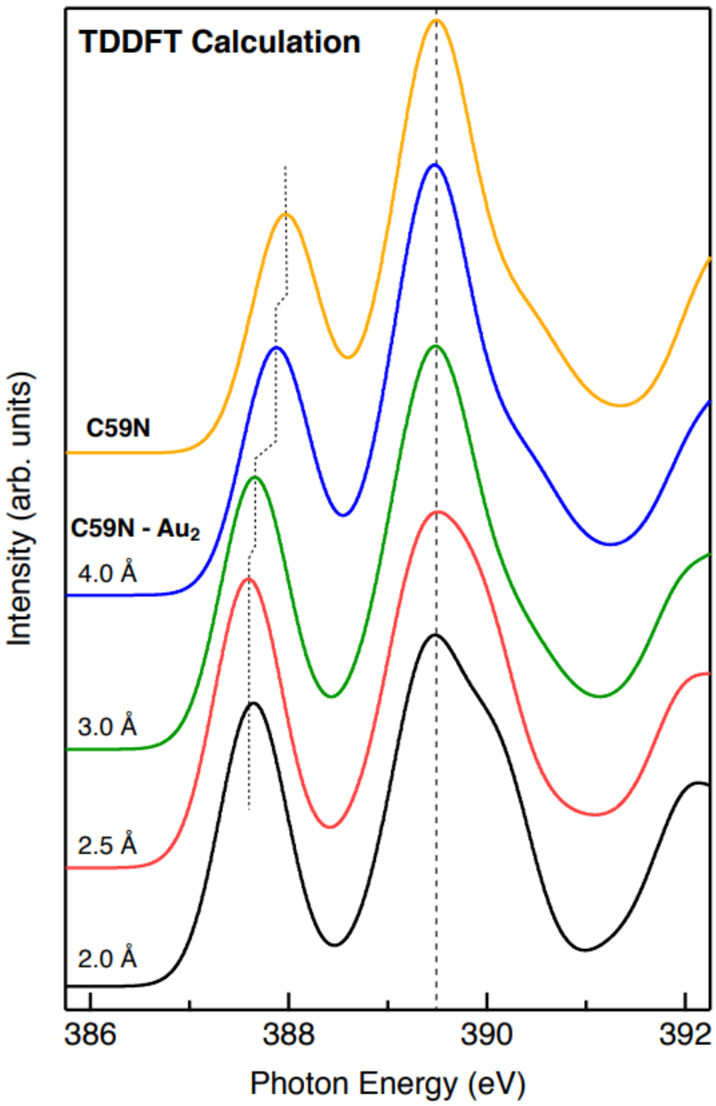

Figure S8. Calculated N 1s NEXAFS spectra of artificial $C_{59}N$-Au_2 complex with different C-Au bond lengths ranging from 2.0 Å to 4.0 Å (labels on the left). Yellow line is the calculation for neutral $C_{59}N^{\bullet}$ in the gas state. All lines are aligned based on the LUMO peak of the calculated $C_{59}N^{\bullet}$ spectrum (dashed line). Dotted line indicates the low-energy shift of the peak due to the $C_{59}N$-Au_2 interaction.

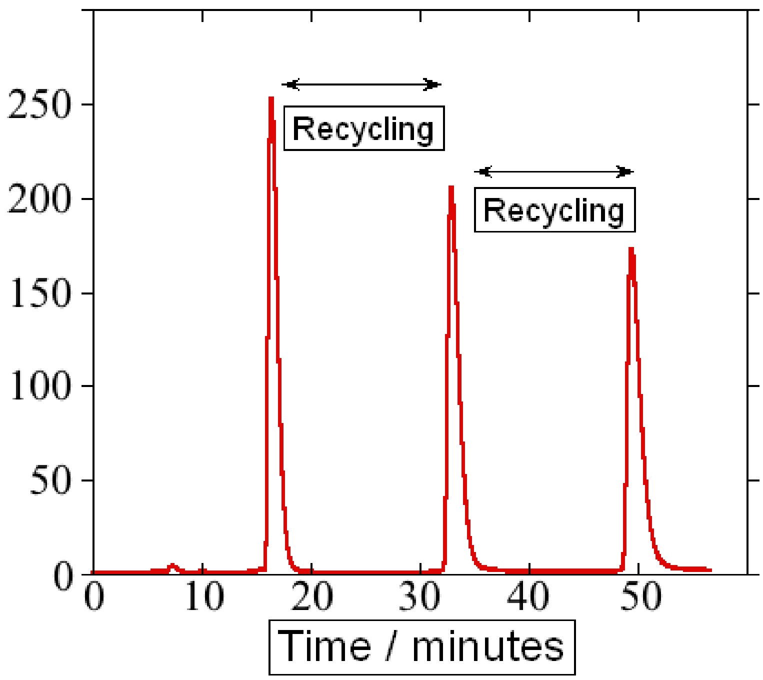

Figure S9. High-performance liquid chromatography (HPLC) trace of $(C_{59}N)_2$ sample. A single peak appears after each recycling proving the sample purity.

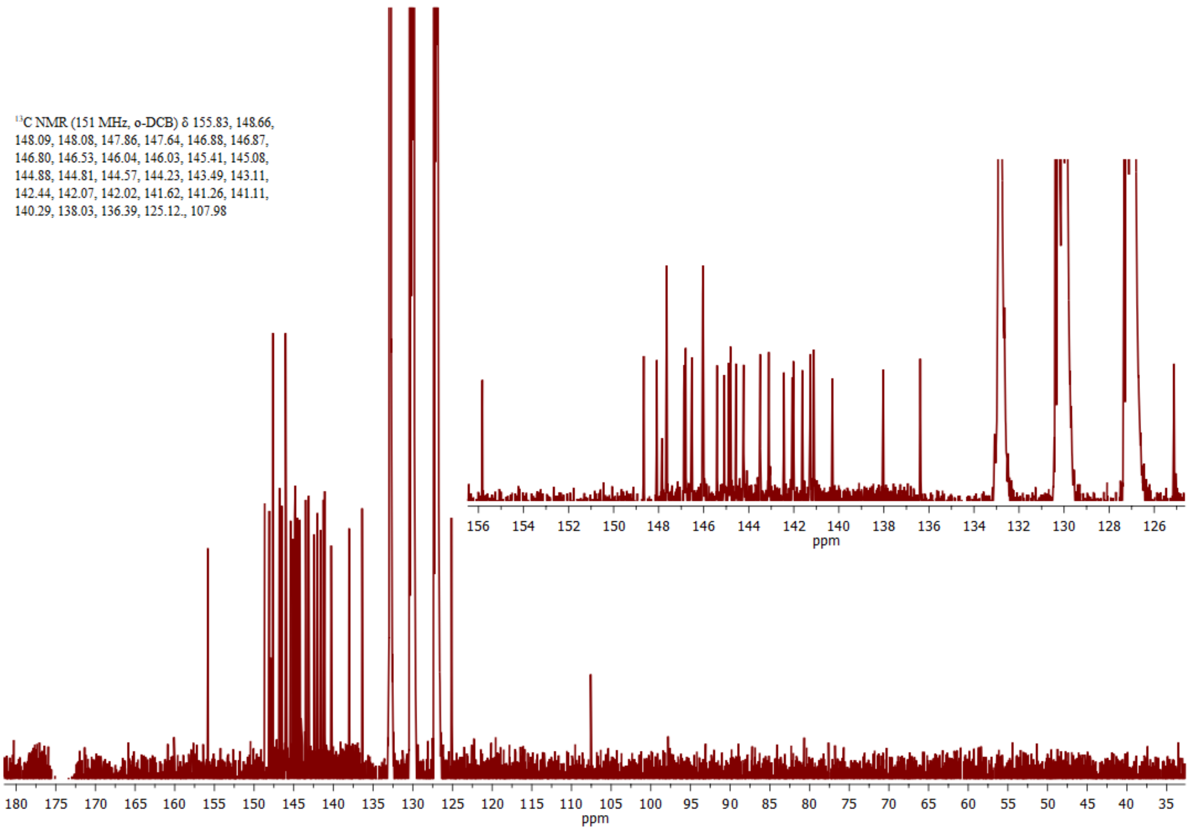

Figure S10. Solution ^{13}C nuclear magnetic resonance (NMR) spectrum of as-prepared $(C_{59}N)_2$ sample, obtained in ortho-dichlorobenzene. In the inset, we show the expanded frequency range between 125 and 157 ppm where characteristic peaks for $(C_{59}N)_2$ were observed.

SA. The $C_{59}N$ radical state within the 1st monolayer

At our deposition temperatures, the $C_{59}N$ are surface mobile and efficiently form extended hexagonally packed monolayer islands attached to Au step edges, as imaged in low-temperature STM (**Fig. 1** in the main text). We below include several experimental and theoretical arguments that support partial retention of the radical character of $C_{59}N$ molecules already within the first monolayer.

(1) Nitrogen tilt angle with respect to the surface: In the lower part of the **Fig. S11a-c** separate N 1s NEXAFS spectra for the photon polarization in TM (p-pol, I_p) and TE (s-pol, I_s) are shown for films with a thickness between 3.5 Å and 7.3 Å. From the linear dichroism of the LUMO (*i.e.* SUMO) and LUMO+1 peak intensity (I_s/I_p) we deduce the orientation of N site within the fullerene cage relative to the surface[1]. The resulting N site orientations are given in terms of average tilt angles (θ) from the surface normal. In the **Fig. S11a** the I_s/I_p=0.2 ratio yields the average nitrogen orientation mainly toward the Au(111) substrate (θ = 30°), which is almost constant throughout the monolayer coverage range (i.e., up to 7.3 Å, in **Fig. S11c**). Such azafullerene orientation clearly demonstrates the interaction of the Au(111) and $C_{59}N$ molecules in the first monolayer. The spectra for the supramonolayer (1+ML) film shown in **Fig. S12b** are substantially less dichroic in agreement with the random $C_{59}N$ radical orientation within the 2nd layer. On the other hand, for the 2 ML film (**Fig. 3** in the main text) I_s/I_p=1.2 proving that N is preferentially oriented in-plane (θ = 60° or 120°) as expected for the $C_{59}N$ in-plane coupling into $(C_{59}N)_2$ dimers. For each film we have also measured the C 1s XPS binding energy, which yields the Au screening shift and serves as an indicator of the film proximity from the Au(111).

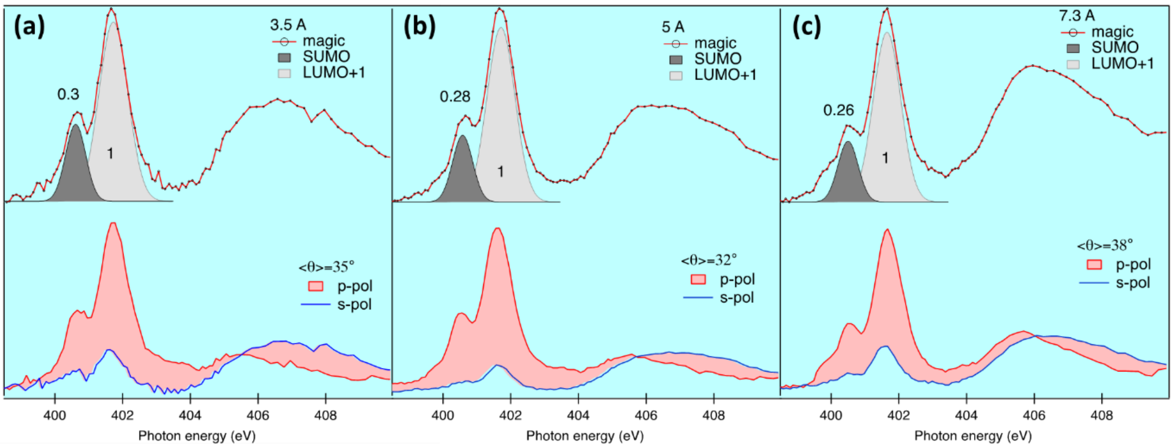

Figure S11. N 1s NEXAFS spectra for increasing $C_{59}N$ coverage of 3.5 Å (a), 5 Å (b), and 7.3 Å (c), respectively. Upper panels: The LUMO peak intensity (400.6 eV) is compared to respective LUMO+1 peak in the "magic angle" spectra. Bottom panels: The N 1s NEXAFS dichroisms (s-pol/I-pol spectra) are shown for each film thickness, indicating that throughout the monolayer the $C_{59}N$ orientation is mostly preserved.

(2) Radical peak intensity (I_{LUMO}) as a function of film thickness: In the upper panel of **Fig. S12** we show the N 1s NEXAFS spectra in the "magic angle" for three characteristic $C_{59}N$ coverages. For the monolayer, the NEXAFS intensity of the radical peak (LUMO at 400.3 eV) relative to LUMO+1 is 24±4%, whereas it is 34% in the supramonolayer radical film and less than 14% in the dimerized 2 ML film.

This proves that on Au(111) the $C_{59}N$ monomers *partially* retain their radical state despite their coupling to Au surface (which dictates the observed N orientation toward the surface as argued above). However, from the present data it is impossible to resolve the nature of the radical state quenching (I_{LUMO} decrease from 34% to 24 %) since Au surface displays numerous sites where $C_{59}N$-Au interaction is particularly strong (monoatomic steps, adatoms/vacancies and specific sites of the Au(111) herringbone reconstruction and possibly also Au sites where $C_{59}N$ adsorbs with its sp^3 carbon of the fullerene cage exactly above the Au atom – see DFT results below).

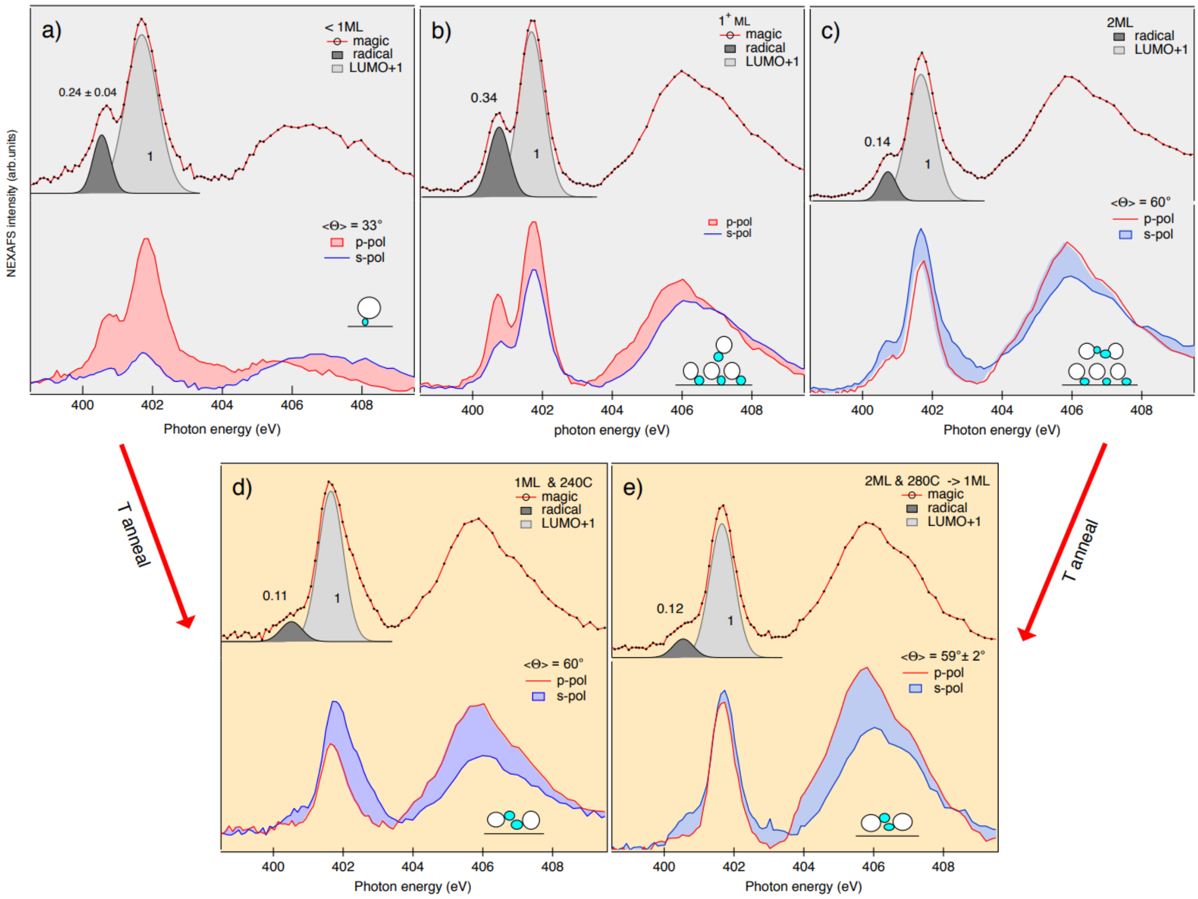

Figure S12. N 1s NEXAFS of $C_{59}N$ film with different thicknesses and their change after thermal annealing: as prepared (a) submonolayer (<1 ML), (b) supramonolayer (1+ ML), (c) complete two layer (2 ML), which are compared to (d) 1 ML film after annealing at 240°C, and (e) 2 ML film after annealing at 280°C. Upper panels: N 1s NEXAFS spectra taken at the "magic angle" with spectral decomposition of LUMO and LUMO+1. The relative intensities of the radical peak (400.6 eV) with respect to the LUMO+1 (401.3 eV) are indicated. Lower panels: linear dichroism of N 1s NEXAFS measured with TM (p-pol) and TE (s-pol) photon polarization. The N angle to the surface normal is given as θ. Visual sketch of the respective orientation is shown in the inset; white and light blue circles represent fullerene cage and the orientation of N site, respectively.

(3) Thermal annealing of $C_{59}N$ layers: Annealing of ML films dramatically affects the radical state of $C_{59}N$ (**Fig. S13d,e**). Namely, when the 1 ML film is thermally annealed at 240°C, the radical peak at 400.6 eV is mostly quenched (<11%) and the overall spectrum then closely resembles that of the in-plane oriented $(C_{59}N)_2$ dimers observed for the 2 ML film (**Fig. 3** in the main text). Importantly, the C 1s XPS still shows that after annealing the sample remains as a monolayer film. In addition, the linear dichroism reflecting the N orientation toward Au(111) is reversed (I_s/I_p=1.2, **Fig. S12a,d**), also confirming that the annealed monolayer here is made of non-radical, in-plane oriented $(C_{59}N)_2$ dimers. **Fig. S12e** shows the thermally annealed 2 ML film which also turns into 1 ML film of $(C_{59}N)_2$ dimers, proving that the 2nd layer dimer adsorption is substantially weaker than that for the $(C_{59}N)_2$ on Au(111). In this case the first monolayer thus becomes fully sacrificial, no longer demonstrating spin. As such this shows that temperature is a useful tool that allows us to control the spin state of this first monolayer.

Finally, the slightly broader radical peak seen in the N 1s NEXAFS for the 1 ML compared to the 1+ ML is consistent with local variations in C-Au distances. In agreement with the calculations and the STM showing some azafullerenes height variation, which depends on the local stacking match with the Au (**Fig. 1b** in the main text).

(4) $C_{59}N$ interaction to the Au(111) surface from DFT calculations: The surface interaction of the first monolayer is next theoretically investigated with DFT calculations for low surface density $C_{59}N$ on Au (25% surface coverage, i.e., avoiding the interaction between neighboring $C_{59}N$ present in the islands). In this case the lowest energy structure has the previously unbonded carbon of the $C_{59}N$ *directly* above a Au atom in the layer below, with the nitrogen atom at approximately 30° inclination to the surface normal. In this case there is the formation of a Au-C sigma bond (s-p character), removing the radical state

from around the Fermi level and replacing it with a bonding and anti-bonding pair deeper into the valence and conduction bands respectively (**Fig. S13a**). The Au-C bond length is 2.2 Å, and the system has no net spin. By taking the difference in total charge density between this system, and the calculated charge density for the $C_{59}N$ and Au calculated separately and then simply summed, we obtain the change in charge density distribution caused by the proximity of the $C_{59}N$ to the Au, which we plot in **Fig. S13b**. This shows a dipolar redistribution of charge around the azafullerene surface associated with the bond formation.

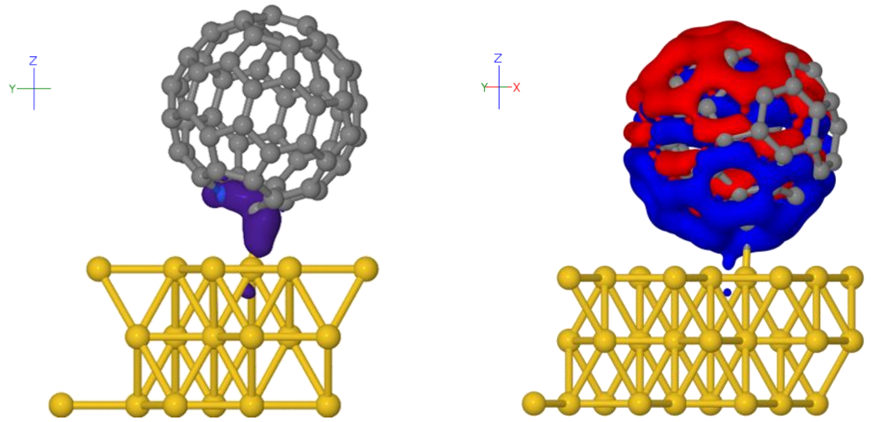

Figure S13. (a) Bonding orbital between $C_{59}N$ and Au when positioned in ground state configuration with the former radical directly above an Au atom, (b) charge density difference induced by proximity of $C_{59}N$ to Au surface, red isosurface corresponding to a decrease of the charge density and blue an increase (see text) as compared to charge density distribution in isolated Au and $C_{59}N$.

In order to investigate the effect of height variation on the system spin, we carried out a sequence of single point energy calculations stepping the azafullerene away from the surface, holding all atoms fixed in the system except for the carbon atom facing the gold surface. In each case the system spin is allowed to fully relax. The result is shown in **Fig.**

S14, showing that the $C_{59}N$ recovers a non-zero partial net spin already when ~0.6 Å further from the surface than its fully relaxed surface bound configuration.

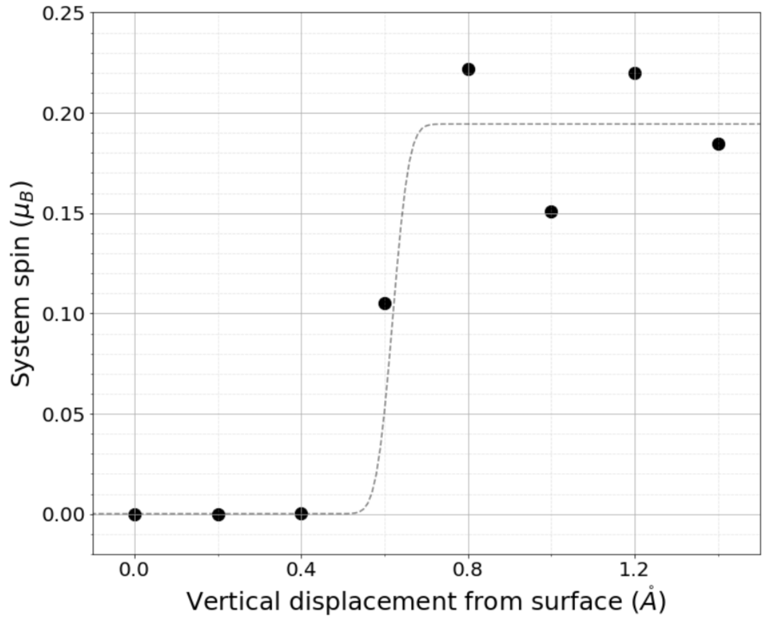

Figure S14. Total system spin (μ_B) as a function of $C_{59}N$ distance from its equilibrium height above the Au surface. All atoms are held fixed except for the carbon atom closest to the surface, spin allowed to fully relax at each point. The dashed line is a guide to the eye.

The experimental STM images in **Fig. 1b** of the main text and in **Fig. S1** show significant variation of about ~1.5 Å in azafullerene distance from the surface. This can be caused by various factors but notably the misfit in the lattice spacing between the azafullerenes and the underlying gold layer, which necessarily imposes that not every $C_{59}N$ can sit directly above an Au atom. Thus in light of the calculations we expect that a weak radical spin will be seen in those further from the surface, as detected in the experimental NEXAFS of the first monolayer. There are some parallels with the Au_2-$C_{59}N$ configuration used for the TDDFT NEXAFS simulations (see **Fig. 3b** in the main text). In this case the Au

forms a non-metallic bound pair and cannot covalently bond to the $C_{59}N$, and the result is the lower energy peak visible in the N 1s NEXAFS associated with the radical on its carbon neighbor, similar to that observed in experiment.

In conclusion, DFT calculations demonstrate that some of the adsorbed $C_{59}N$ molecules in the first monolayer will strongly bind to Au and lose their radical character. However, mismatch between the azafullerene and Au lattices will cause many of the $C_{59}N$ molecules to move away from the Au, weakening their bonding and recovering a weak radical state, as detected in the N 1s NEXAFS.

In summary, we can now provide a full and complete description of the behavior of the first azafullerene layer on Au(111). In the very early deposition stages, it gives non-radical $C_{59}N$ that form a weak sigma bond to underlying Au. These molecules are highly surface mobile, rapidly forming a close-packed monolayer islands primarily attached to surface steps on the Au. The slight broadening of the N 1s NEXAFS peak comes from the variation in Au-C distances caused by the lattice mismatch between Au and $C_{59}N$. Lifting numerous $C_{59}N$ monomers away from the surface for these entities blocks the possibility of covalent Au-C bonding. As a result, a radical signal is experimentally observed in N 1s NEXAFS, with the linear dichroism demonstrating that the preferred orientation of N toward the surface is still preserved. Heating the monolayer to 240°C allows the fullerenes to overcome an activation barrier for rotation and dimerization, resulting in an almost complete radical quenching as the contact layer of radical monomers undergoes a phase transition to stable monolayer of $(C_{59}N)_2$ dimers, with the inter-fullerene C-C bond lying parallel to the surface. These results demonstrate that 1 ML is rather inhomogeneous where some of the adsorbed azafullerene molecules retain their radical state and the others do not.

Reference:

1 Stöhr, J. NEXAFS spectroscopy; Springer: Berlin, 1992; Vol. 25.
